# Evaluation of Etiologies in Evisceration as Rare Cases: A 10-Year Single-Center Experience in the East Mediterranean Region of Türkiye

**DOI:** 10.3390/jcm14103601

**Published:** 2025-05-21

**Authors:** Burak Ulaş, Altan Atakan Ozcan, Burak Mete, Hakan Demirhindi, Merve Ademoğlu Gök, Hülya Binokay

**Affiliations:** 1 Department of Ophthalmology, Faculty of Medicine, Çukurova University, 01330 Adana, Türkiye; bulas@cu.edu.tr (B.U.); altanoz@cu.edu.tr (A.A.O.);; 2 Department of Public Health, Faculty of Medicine, Çukurova University, 01330 Adana, Türkiye; demirhindi@cu.edu.tr; 3 Department of Biostatistics, Faculty of Medicine, Çukurova University, 01330 Adana, Türkiye; hbinokay@cu.edu.tr

**Keywords:** evisceration, eye removal, endophthalmitis, ocular trauma

## Abstract

**Objectives**: Evisceration is a rarely performed surgical procedure, and literature information regarding the characteristics of patients undergoing this surgery is limited. This study aims to evaluate the demographic features and etiological factors of patients who underwent evisceration surgery in a tertiary clinic over 10 years. **Methods**: This descriptive study comprised the data of 134 patients who underwent evisceration surgery at the Department of Ophthalmology, Faculty of Medicine, Çukurova University, Adana, Türkiye, between 2011 and 2022. Demographic data of all patients, including age, sex, surgical indication, etiology, primary disease location, pathology results, and follow-up periods, were recorded and analyzed. **Results**: The mean age of the 134 patients included in the study was 56.18 ± 22.71 (min: 8–max: 91), with a male-to-female ratio of 65.2% to 34.8%. Evisceration etiologies included trauma (37%), endophthalmitis (37%), absolute blind eye (12.6%), and spontaneous perforation (11.9%). Endophthalmitis cases were more common in older ages and females, trauma patients in younger ages and males, and spontaneous perforation in older ages and males. Progression to panophthalmitis was observed in 6.6% of all cases, and all were found to originate from endophthalmitis. Culture growth was positive in 18.5% of the cases, with the most commonly grown microorganisms in culture being *Pseudomonas aeruginosa*, *Streptococcus dysgalactiae* and *Aspergillus fumigatus*. *Escherichia coli* and *Staphylococcus epidermidis* rates were significantly higher in cases progressing to panophthalmia. Acute inflammation was more prevalent in cases of endophthalmitis and spontaneous perforation, while chronic inflammation was in cases of trauma and absolute blind eyes. **Conclusions**: Trauma was the main etiology of evisceration in young males and endophthalmitis in older females. Considering trauma prevention measures is important for public health in terms of eyeball saving.

## 1. Introduction

Evisceration is a surgical technique in which the contents of the eyeball are removed while leaving the scleral shell in the orbita [1]. Evisceration surgery is usually performed for a painful blind eye due to various underlying etiologies [2]. Globally, the most common indications of evisceration are endophthalmitis and trauma [3,4]. The epidemiologic and etiologic characteristics of eviscerations vary according to geographic regions and socioeconomic levels [2,3,4,5,6]. The indications and approaches for eye removal can vary based on the level of development of countries, e.g., trauma is the predominant indication in developed countries, but infectious causes tend to be more prominent in developing countries [2,3,4,5,6].

The purpose of evisceration surgery is to remove the painful, blinded eye, ensure adequate comfort, restore lost volume, and achieve both functional and cosmetic improvements [7]. The loss of an eye is one of the most traumatic events for people of all ages. Although the removal of the eyeball is a difficult decision for both the patient and the ophthalmologist, it is considered the final option in certain clinical circumstances [8].

Rasmussen et al. [9] indicated that a painful blind eye associated with eye surgery was the most predominant cause of evisceration in Denmark. In another study from France, Genevois et al. [10] reported trauma as the predominant cause of evisceration surgery. A literature review revealed very few studies addressing evisceration in Türkiye, accompanied by a lack of multidisciplinary clinicodemographic and clinical data [11,12].

The aim of this study was to evaluate the clinicodemographic characteristics and etiological factors in patients who underwent evisceration surgery in a multidisciplinary tertiary care university clinic in Southern East Mediterranean Türkiye and to contribute to the Turkish data on evisceration surgery.

## 2. Materials and Methods

**Figure 1:**
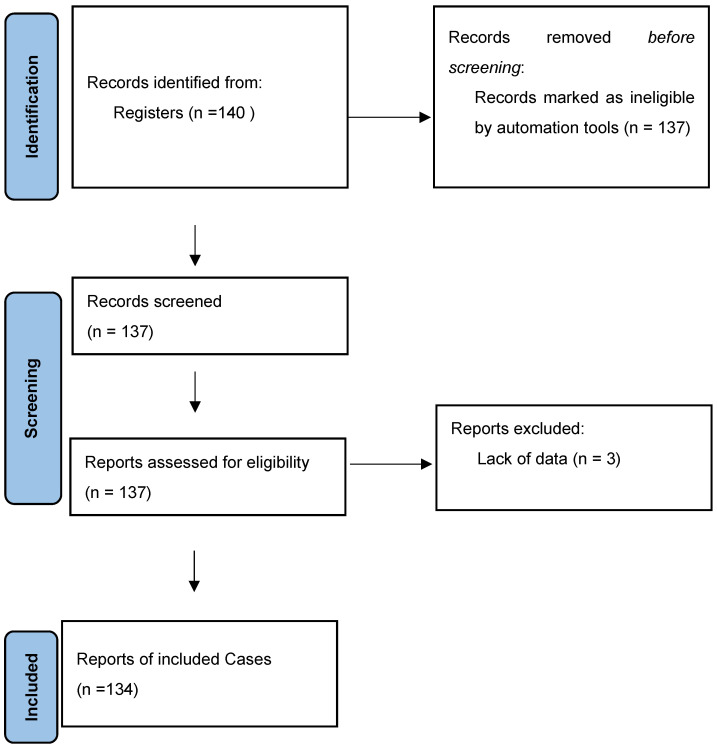
Flow chart for selection of patients.

This descriptive study comprised 134 patients (with a total of 135 eyes) who had undergone an evisceration surgery between 2011 and 2022 at the Department of Ophthalmology, Faculty of Medicine, Çukurova University, Adana, Türkiye. The study can be defined as a large-scale study, as it also included Syrian immigrants from Northern Syria in the southern regions of Türkiye. Ethical approval was obtained from the local ethics committee of the Faculty of Medicine at Çukurova University (Appl. no: 8.12.2023-139-39), and the study adhered to the tenets of the Declaration of Helsinki. The following inclusion criteria were applied: being 18 years old or older, and having a surgical eye removal by evisceration, whatever the indication. The exclusion criteria were loss of follow-up of the patient before three months had passed (the time required for patients to complete wound healing and also the period during which they are at risk for complications) and lack of data. Medical records were evaluated regarding surgical indication of evisceration, clinicodemographic data, mean duration of follow-up, and follow-up clinical features. The selection procedure is presented in Figure 1.

### 2.1. Surgical Technique

In all surgical patients, a 360-degree conjunctival peritomy parallel to the limbus was performed using Westcott conjunctival scissors. The Tenon capsule and conjunctiva were dissected up to the insertions of the rectus muscles from the sclera. Entry into the anterior chamber was made from the limbus using an 11-blade scalpel. Keratectomy was performed using corneoscleral scissors. The uvea was separated from the scleral spur, and the contents of the globe were completely evacuated using an evisceration spatula. Hemostasis was achieved. Absolute alcohol-soaked cotton was swabbed within the scleral cavity to denature any remaining uveal tissues. The scleral cavity was irrigated with a balanced salt solution and an antibiotic solution. After extending the anterior scleral incisions from the 3 o’clock to the 9 o’clock positions to the optic nerve, an appropriate orbital implant was placed. The scleral flaps were sutured using 6/0 Vicryl in an overlapping fashion. The Tenon capsule was closed individually, and the conjunctiva was closed continuously using 7/0 Vicryl. After applying antibiotic ointment, a conformer was inserted prior to the application of tight bandaging on the patient. All surgeries were performed by two experienced oculoplastic surgeons (BU, AAO).

### 2.2. Data Collection

The patients were pre-screened during the preoperative visit to determine whether there was an indication for evisceration surgery. All patients underwent a comprehensive ophthalmologic examination during the preoperative visit, including a measurement of the best corrected visual acuity, a slit lamp examination and fundus examination in both eyes. Then, the patients were the included in the study from the time of the surgery. Patients were informed about the evisceration procedure prior to the operation, and consent was obtained from all patients. All patients received general anesthesia. After surgery, the patients were followed up according to the recommendations of the surgeons (BU, AAO). Following surgery, broad-spectrum oral and topical antibiotic treatments were initiated, and patients were closely monitored with tight bandaging for two days. After surgery, patients were scheduled for follow-up examinations at week 1, month 1, month 3, month 6, year 1, and annually thereafter.

The following data were recorded: patients’ age, sex, eye lateralization, etiology, duration of visual loss, duration of hospitalization, microbiologic results, follow-up durations, post-operative pain and complications. Microbial cultures obtained from pre-operative eye contents from vitreus and anterior chamber, if necessary (endophthalmitis), were documented and categorized. The post-surgical pathology reports of all patients were evaluated and recorded.

### 2.3. Statistical Analysis

The data were analyzed with IBM SPSS Statistics for Windows, version 20.0 (IBM Corp., Armonk, NY, USA). The normality was determined with the Shapiro–Wilk test. Parametric tests were used for normally distributed data where continuous variables were presented as mean and standard deviation. Non-parametric tests were used for data not normally distributed, and results are presented as median and interquartile range (IQR). The chi-square test was used to analyze categorical variables, and results are presented as numbers and percentages. Kruskal–Wallis test and Pearson’s chi-square test were used in the analyses. In chi-square analyses, the exact test was preferred when more than 20% of the expected values were below 5 or the minimum expected value was below 1. A *p* < 0.05 was considered statistically significant.

## 3. Results

**Figure 2:**
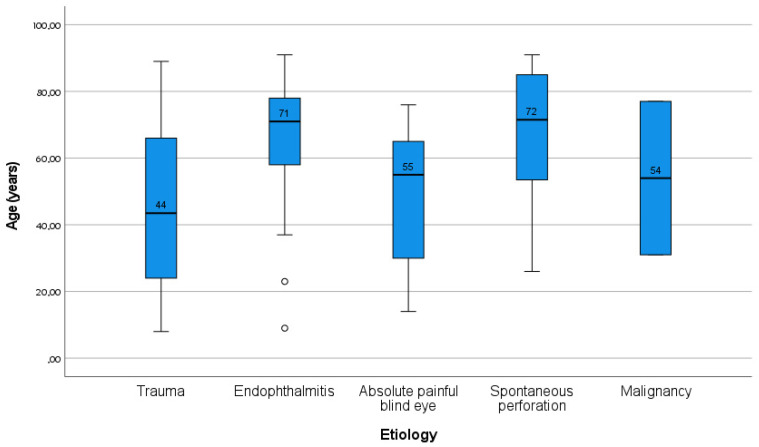
Age distribution according to etiology (box-plot graphic).

The mean age of the 134 patients included in our study was 56.18 ± 22.71 years (min. 8–max. 91), with a male-to-female ratio of 88 (65.2%) to 47 (34.8%). The etiologies of evisceration were trauma with a rate of 37% (n = 50), endophthalmitis 37% (n = 50), abscess 12.6% (n = 17), spontaneous perforation 11.9% (n = 16), and malignancy 1.5% (n = 2). The demographic and clinical characteristics of the patients according to the etiologies are given in Table 1. It was found that the median age was highest in patients with endophthalmitis, while it was the lowest in trauma patients (Figure 2). In addition, endophthalmitis cases were more common in females, and trauma cases in men. While all cases of panophthalmitis were found to have originated from endophthalmitis, only 18% of the endophthalmitis patients progressed to panophthalmitis, and this etiological difference was statistically significant. Microbiological culture growth rates were higher in endophthalmitis and spontaneous perforation cases compared to the trauma group. Amoeba growth was observed in only one trauma case (Table 1).

The microbiological culture results revealed that the most commonly grown bacterial microorganisms were *Pseudomonas aeruginosa* and *Streptococcus dysgalactiae*, while *Aspergillus fumigatus* was observed in the first rank among fungal microorganisms (Table 2).

## 4. Discussion

Ocular evisceration is a surgical procedure for end-stage ophthalmological diseases which are not curable with medical or surgical treatments [1,2]. Evisceration is a minimal procedure in eye removal surgeries, constituting mainly a cosmetic procedure. Eye removal surgeries are performed as a last option to treat ophthalmic conditions such as a painful blind eye, endophthalmitis, or globe trauma [3,4,5]. The loss of an eye is a significant life event and important condition that has both psychological effects and is a public health problem [6,7,8]. In this study, we aimed to evaluate the etiology, epidemiology and referral reasons of patients who underwent evisceration surgery in a tertiary university clinic.

In the present study comprising 135 patients, endophthalmitis (37%) and trauma (37%) were the most common etiologic factors, with a same rate. Similar to other developing countries, our study revealed that infections and trauma were significantly prominent reasons for destructive eye surgeries in Türkiye [3,4,5,6,7,8]. Furthermore, the rate of trauma among patients aged 30 years and younger was 67.9%. We believe that the higher number of evisceration surgeries due to endophthalmitis and trauma can be attributed to our hospital’s status as a referral center in the region.

Dada et al. [13], who evaluated 164 patients in Northern India, reported that panophthalmitis ranked first among indications for evisceration, accounting for 78%. The authors attributed this high frequency of infectious causes to ophthalmic preparations not prepared under sterile conditions, leading to corneal ulceration [13]. In our study, the rate of panophthalmitis was 6.6%. These findings further support the evidence that epidemiologic and etiologic characteristics of eviscerations vary according to geographic regions and socioeconomic levels.

The indications of evisceration surgery may vary depending on the economic situations of the countries. In developed countries, the two most common causes of eye removal surgeries are tumors and painful blind eyes, whereas they are trauma and endophthalmitis in developing countries [13,14,15,16,17]. Similar studies in the literature aimed to determine the etiological factors contributing to evisceration. In their research conducted in Denmark, Hansen et al. [18] identified trauma, endophthalmitis, glaucoma, and inflammation as the most common etiologic factors, in descending order, leading to evisceration. They also reported that, similarly to our findings, patients of evisceration resulting from trauma were predominantly males aged 30 years and younger. Rasmussen et al. [9] reported that the most common cause of eye removal procedures conducted in Denmark was painful eyes and malignities. In another study conducted in Turkey by Balta et al. [12], the most common reasons for evisceration were trauma (60%), glaucoma (13%), and postoperative endophthalmitis (12%). De Gottrau et al. [19] stated that the most important causes of eye removal were trauma and tumors in Germany.

Ocular trauma is a preventable cause of eye injury and, along with infectious eye diseases, is one of the leading reasons for evisceration in developing countries [20,21]. Traumatic indications for enucleation or evisceration were more prevalent among men than women [21]. This is consistent with the majority of published research, which indicates that men are more prone to experiencing trauma [20,21]. Taking measures specifically tailored to age, sex, and different risk groups will help reduce eye injuries and consequently decrease the need for evisceration surgeries due to trauma. In Türkiye, trauma is the most common cause in studies [11,12]. This is a major public health problem of young and economically productive ages. Balta et al. [12] reported that, in their series of trauma group undergoing evisceration surgery in Türkiye, approximately 65% of the patients were male, and their mean age was 42, which could be mentioned as the most economically productive period in their life. This means that trauma, the most important preventable etiological factor, is a major public health problem in developing countries. It is important to take some precautions in this regard, such as increasing socio-cultural and economic levels, raising awareness in families, taking protective measures in workplaces, complying with traffic rules and raising public awareness [20,21].

Painful blind eye is a difficult and debilitating condition that significantly impacts patients’ quality of life, being observed in one of every ten people with blindness [22,23]. This condition is characterized by a visual acuity of counting fingers or worse, with no hope of regaining function, and is accompanied by chronic pain and discomfort lasting at least four weeks [22]. Painful blind eye is defined as a terminal condition where vision is unsalvageable. Painful blind eye conditions in Africa significantly impact the quality of life and well-being of the affected individuals, their families, and their communities [22,23]. The decision to remove an eye can be difficult when it appears anatomically normal. Therefore, emotional and psychological preparation gains priority when considering this approach [24]. Understanding the patient’s expectations and beliefs from the outset is essential for effective preparation. The literature indicates that there is no difference between evisceration and enucleation surgeries in terms of the risk of sympathetic ophthalmia and postoperative discomfort in these patients [25]. In our study, 17 (12.6%) patients underwent evisceration due to a painful blind eye.

Endophthalmitis is an inflammatory process affecting the globe tissues, which is associated with infection [26]. The most common risk factors of endophthalmitis are trauma, intraocular surgery, keratitis, and hematogenous dissemination of infectious microorganisms to the globe [18,19,20,21]. Despite medical and surgical treatments, endophthalmitis can sometimes progress to the point where the globe must be removed by evisceration [26]. Severe cases of endophthalmitis may require an evisceration procedure. In the present study, endophthalmitis was present in the etiology of evisceration at a rate of 37%. Additionally, 18% of patients with endophthalmitis progressed to panophthalmitis. Merly et al. [17] reported that the progression of cases to panophthalmitis increased the need for evisceration of the globe. In our clinical experience and study, we observed that the need for evisceration was higher in cases progressing to panophthalmitis. When we analyzed the culture results obtained from evisceration patients with endophthalmitis, as well as the pathology results, we found that the most commonly isolated pathogen was *Pseudomonas aeruginosa*, detected in nine patients, accounting for 6.6% of cases, followed by *Escherichia coli*, *Streptococcus pneumoniae*, and *Aspergillus*, which were identified in two patients each, representing 1.7%. Merly et al. [17] stated that, in their study, positive microbiology isolates were present in 31 eyes (44.93%), where 23 isolates (73.3%) were gram-positive bacteria, seven isolates (23.3%) were gram-negative bacteria, and one was fungal (3.2%). In our study, pathologic evaluation revealed chronic inflammation as the most frequently observed pathologic finding, accounting for 65.9%, while acute inflammation accounted for 29.6%. Ahmad et al. [2] stated in their study from the U.S.A. that they had to perform evisceration in 9.88% of endophthalmitis cases. They reported that 37.5% of endophthalmitis cases were post-procedural, 21% were post-traumatic, and 10% were of endogenous etiologies [2]. Ozdemir et al. [27] reported that in the microbiological evaluation results of patients who underwent evisceration after penetrating keratoplasty, *Aspergillus flavus* was found in three patients, *Candida albicans* in one patient, *Staphylococcus aureus* in three patients, *Bacillus cereus* in one patient, and *Pseudomonas aeruginosa* in one patient. When the studies in the literature were evaluated, the microbiological and pathological evaluations of patients who underwent evisceration varied according to their etiology [26,27].

The median age of eye evisceration in our population was 56 years, which is close to that in developed countries and higher when compared to some developing countries [2,3,4,5]. In the study conducted by Yousuf et al. [14] from the U.S.A., the mean age of the patients in their evisceration series of 54 patients was 51 ± 29 years, with 61% being male. In a series of 69 patients reported by Merly et al. [17] from Puerto Rico, the mean age was 70 years, and 59% of the patients included in the study were male. In their study examining trauma-related evisceration cases reported from China, Jiang et al. [20] reported that 89% of the participants were adults (over 18 years of age), and 40% were between 18 and 30 years of age. In their study conducted in Hungary, Toth et al. [28] reported that the average age of patients who underwent evisceration/enucleation due to corneal ulcers was 66.4, and 61% of the cases were female. Therefore, different studies in the literature indicate that demographic data (age, sex) of the patients undergoing evisceration vary according to the etiology of evisceration and, in addition, according to the geography of residence and the developmental level of the countries.

The most common complications of evisceration surgery are wound dehiscence and implant extrusion [1,29]. Sympathetic ophthalmia is a major complication of evisceration surgery, presenting in the form of granulomatous panuveitis that occurs following surgical or non-surgical ocular trauma in one eye [29]. It constitutes one of the most feared postoperative complications, and it can occur anywhere between five days and 66 years after the procedure [29]. Although sympathetic ophthalmia is not noted as a concern, there seems to be a belief that evisceration poses a much higher risk for sympathetic ophthalmia compared with enucleation. However, in the literature, the evisceration procedure is said to pose a very low risk of sympathetic ophthalmia [2,3,4,5,6]. The selection of the surgical procedure varies based on the geographic area and the surgeon’s individual preference. Previous research suggested that the preference for enucleation to prevent sympathetic ophthalmia should be reevaluated due to its low risk and the advantages of evisceration in terms of eye movement and cosmetic outcomes [3,4,5,6,7,8]. In our study, there were no recorded instances of sympathetic ophthalmia development during the follow-up period. Similarly, recent studies reported the risk of sympathetic ophthalmia following evisceration to be very low [1,2,3,4,5,6,7,8].

Eagle et al. [30] reported seven cases inadvertently eviscerated with intraocular malignancy. In their study, they stated that an adequate preoperative evaluation is mandatory before evisceration surgery. In the same study, the authors also reported that preoperative imaging did not totally exclude the possibility of inadvertently eviscerating an eye with occult melanoma [30]. As stated in the literature, this means that sometimes an evisceration operation can be performed on eyes with intraocular tumors [30]. In our study, uveal melanoma was diagnosed in the evisceration samples of two patients who underwent evisceration with the diagnosis of painful blind eye.

Preventing eye loss can be achieved through public education, enforcement of health and safety standards, creation of safe living and working environments, early detection of eye diseases, and prompt treatment by well-trained healthcare professionals. Vision loss may be avoided with community awareness, proper implementation of safety regulations, safe conditions at home and in the workplace, early diagnosis of ocular diseases, and timely intervention by skilled medical personnel. Through health education, strict safety measures, and early identification of eye conditions followed by appropriate care from qualified professionals, the risk of vision impairment can be significantly reduced. Public awareness campaigns, strong health and safety policies, safe environments, and prompt, expert medical response to early signs of eye disease can help prevent loss of sight. Loss of vision can be prevented by educating the public, enforcing safety rules, maintaining secure environments, and ensuring early and proper treatment of eye disorders by trained healthcare providers [5].

There are some limitations to our study. First of all, the retrospective nature of the study and the possibility of selection bias should be mentioned. Secondly, since our hospital is a multidisciplinary tertiary university health center, some of the follow-ups of these patients could not be completed, but they provided valuable findings due to the wide variety of etiologies of the patients. Despite the limitations, the present study is very important because of the rarity of this procedure, evisceration. It is one of the largest retrospective studies reported from Türkiye, and it is valuable for enriching the existing knowledge about evisceration surgery.

## 5. Conclusions

According to the results of our study, endophthalmitis is the main cause of evisceration in females, while trauma and other causes are more common in males. Trauma cases are observed at younger ages. Considering the sex and age distribution, identification of risk factors may help to eliminate preventable causes and reduce the need for evisceration procedures. Eye loss can be prevented by raising public awareness, applying strict health and safety regulations, ensuring safe work and living conditions, detecting eye diseases early, and providing timely care from skilled healthcare professionals. Multi-center and national studies are needed, and studies focusing on the changing trend in clinico-demographics and etiology are needed.

## Figures and Tables

**Table 1:** Comparison of clinical and demographic characteristics according to the etiology of evisceration.

	Aetiological Groups *n* (Column %) or Median [*IQR*]					*p*
Variables	Trauma *n* = 50	Endophthalmitis *n* = 50	Absolute Blind Eye *n* = 17	Spontaneous Perforation *n* = 16	Malignancy *n* = 2	
Age (years)	43.5 (42.5) ^a,b^	71 (20) ^a^	55 (39.5)	71.5 (33.75) ^b^	54 (N/A)	**<0.001**
Follow-up period (month)	3 (5)	4.5 (13)	10 (33)	6 (21.25)	3 (0)	0.050
Sex						
Male	37 (74)	26 (52)	13 (76.5)	10 (62.5)	2 (100)	**0.049**
Female	13 (26)	24 (48)	4 (23.5)	6 (37.5)	0 (0)	
Affected eye						
Right	27 (54)	26 (52)	12 (70.6)	10 (62.5)	1 (50)	0.754
Left	23 (46)	24 (48)	5 (29.4)	6 (37.5)	1 (50)	
Panophthalmitis						
None	50 (100)	41 (82)	17 (100)	16 (100)	2 (100)	**0.009**
Present	0	9 (18)	0	0	0	
Growth in culture						
None	48 (96)	32 (64)	17 (100)	11 (68.8)	2 (100)	**<0.001**
Present	2 (4)	18 (36)	0 (0)	5 (31.3)	0 (0)	
Culture results						
Bacteria	1 (50)	16 (88.9)	NA	3 (60)	NA	**0.032**
Fungus	0 (0)	2 (11.1)	NA	2 (40)	NA	
Amoeba	1 (50)	0	NA	0	NA	
Pathology						
Acute suppurative inflammation	13 (26)	20 (40)	2 (11.8)	5 (31.3)	0 (0)	**<0.001**
Chronic inflammation	37 (74)	29 (58)	12 (70.6)	11 (68.8)	0 (0)	
Other osseous metaplasia, dystrophic calcification, metastasis, haemorrhage-congestion	0 (0)	1 (2)	3 (17.6)	0 (0)	2 (100)	
Sympathetic ophthalmia	0	0	0	0	0	N/A
Total	50 (100)	50 (100)	17 (100)	16 (100)	2 (100)	

Symbols ^a,b^ indicate post hoc analysis results where differences exist between different lettered groups; bold values indicate statistical differences; IQR, inter-quartile range; N/A, not applicable.

**Table 2:** Distribution of microorganisms grown in microbiological culture.

Growth Result	*n*	%
None	110	81.5
*Pseudomonas aeruginosa*	9	6.6
*Streptococcus dysgalactiae*	3	2.1
*Aspergillus fumigatus*	2	1.5
*Escherichia coli*	2	1.5
*Staphylococcus epidermidis*	2	1.4
*Streptococcus pneumoniae*	2	1.5
*Acanthamoeba*	1	0.7
*Candida albicans*	1	0.7
*Fusarium*	1	0.7
*Proteus mirabilis*	1	0.7
*Staphylococcus hominis*	1	0.7
Total	135	100.0

## Data Availability

The data presented in this study are available on request from the corresponding author due to (specify the reason for the restriction).
